# A Necroptosis-Related lncRNA Signature Predicts Prognosis and Indicates the Immune Microenvironment in Soft Tissue Sarcomas

**DOI:** 10.3389/fgene.2022.899545

**Published:** 2022-06-20

**Authors:** Binfeng Liu, Zhongyue Liu, Chengyao Feng, Chao Tu

**Affiliations:** ^1^ Department of Orthopaedics, The Second Xiangya Hospital, Central South University, Changsha, China; ^2^ Hunan Key Laboratory of Tumor Models and Individualized Medicine, The Second Xiangya Hospital of Central South University, Changsha, China

**Keywords:** necroptosis, lncRNAs, prognosis, immune, soft tissue sarcoma

## Abstract

**Background:** The necroptosis and long noncoding RNA (lncRNA) are critical in the occurrence and development of malignancy, while the association between the necroptosis-related lncRNAs (NRlncRNAs) and soft tissue sarcoma (STS) remains controversial. Therefore, the present study aims to construct a novel signature based on NRlncRNAs to predict the prognosis of STS patients and investigate its possible role.

**Methods:** The transcriptome data and clinical characteristics were extracted from The Cancer Genome Atlas (TCGA) and Genotype-Tissue Expression database (GTEx). A novel NRlncRNA signature was established and verified by the COX regression analysis and least absolute shrinkage and selection operator (LASSO) regression analysis. Subsequently, the K-M survival analysis, ROC, univariate, multivariate Cox regression analysis, and nomogram were used to evaluate the predictive value of the signature. Also, a variety of bioinformatic analysis algorithms explored the differences between the potential mechanism, tumor immune status, and drug sensitivity in the two-risk group. Finally, the RT-qPCR was performed to evaluate the expression of signature NRlncRNAs.

**Results:** A novel signature consisting of seven NRlncRNAs was successfully established and verified with stable prediction performance and general applicability for STS. Next, the GSEA showed that the patients in the high-risk group were mainly enriched with tumor-related pathways, while the low-risk patients were significantly involved in immune-related pathways. In parallel, we found that the STS patients in the low-risk group had a better immune status than that in the high-risk group. Additionally, there were significant differences in the sensitivity to anti-tumor agents between the two groups. Finally, the RT-qPCR results indicated that these signature NRlncRNAs were abnormally expressed in STS.

**Conclusion:** To the best of our knowledge, it is the first study to construct an NRlncRNA signature for STS. More importantly, the novel signature displays stable value and translational potential for predicting prognosis, tumor immunogenicity, and therapeutic response in STS.

## Introduction

Soft tissue sarcoma (STS) is a class of rare, highly aggressive, and heterogeneous malignancies derived from connective tissue (Bourcier et al., 2019). There are more than 70 histological subtypes of STS, accounting for around 1% of adult malignancies and may occur in various body locations (Yang et al., 2019). Although STS has a lower incidence than other malignancies, its incidence is relatively more frequent in adolescents, accounting for 10% of patients in this age group (Wibmer et al., 2010; Hung et al., 2014). Currently, the main treatment strategies for STS include surgical resection, radiotherapy, and chemotherapy, and the 5-years survival rate after diagnosis ranges from 55.5 to 56.5% (Bessen et al., 2019; Kim et al., 2019; Kollár et al., 2019). However, the 5-years survival rate of advanced STS patients dramatically dropped to 27.2% (Kim et al., 2019). Meanwhile, STS is characterized by solid invasiveness, high recurrence, and high metastasis, which do incredible harm to human health worldwide. Therefore, it is crucial to find reliable biomarkers for early diagnosis, risk assessment, and prognosis prediction of STS patients.

As a novel programmed cell death (PCD), necroptosis was first described in 2005 by Degterev et al.(Degterev et al., 2005). Necroptosis is a caspase-independent form of cell death, usually manifesting morphological features of necrosis, which was mediated by receptor-interacting kinase 1 (RIPK1), receptor-interacting kinase 3 (RIPK3), and mixed lineage kinase-like (MLKL) (Marshall and Baines, 2014). Similar to other forms of PCD, such as pyroptosis, necroptosis has recently been proposed as a vital factor in regulating tumorigenesis and progression. For instance, the downregulation of RIP3 in acute myeloid leukemia (AML) could reduce hematopoietic cell death, which was associated with the development of AML (Nugues et al., 2014). The significant role of necroptosis in tumor development, necrosis, metastasis, and tumor response, indicates the potential of targeting necroptosis as a new tumor therapy.

Long noncoding RNA (lncRNA) is a class of non-coding RNA longer than 200 nucleotides in length and originates from the non-coding region of the genome (Beermann et al., 2016). LncRNAs were regarded as one of the most sensitive and specific cancer biomarkers. They can regulate the occurrence and development of cancers at various levels, such as transcriptional, post-transcriptional regulation, and epigenetics (Zheng et al., 2019; Jiang et al., 2020). For instance, lncRNA KCNQ1OT1 promotes growth and the Warburg effect in osteosarcoma via the miR-34c-5p/ALDOA axis (Shen et al., 2020). Additionally, accumulating reports have revealed that necroptosis-related lncRNAs (NRlncRNAs) can be used for the prognosis prediction of the tumor, which develops new therapeutic targets for future tumor treatment. For example, Zirui Zhao et al. constructed an NRlncRNA signature and proved it could predict prognosis and improve individual therapy for gastric cancer (Zhao Z. et al., 2021). However, the prediction value and the potential functional role of NRlncRNAs for STS remain unknown. Therefore, it is urgent to explore the relationship between NRlncRNAs and the prognosis of STS and their possible regulatory mechanisms in STS progression.

In the present study, we performed differential analysis and Pearson Correlation analysis based on existing public data to obtain the NRlncRNAs expression matrix in STS. In parallel, a novel NRlncRNA risk prognosis signature was constructed to further reveal the effect of NRlncRNAs on the prognosis of patients with STS. Additionally, we then divided the patients into high- and low-risk groups according to the median risk scores and conducted GSEA, infiltration analysis, and drug sensitivity analysis between the two different risk groups. Herein, our study first comprehensively explored the NRlncRNAs in STS and clarified the preliminary role of NRlncRNAs in the tumor microenvironment and prognosis of STS, which provides not only novel insights into the molecular mechanism of necroptosis in STS but also a theoretical basis for individualized therapies based on NRlncRNAs.

## Materials and Methods

### Data Acquisition

The expression data of each STS sample and normal control tissue samples were obtained from The Cancer Genome Atlas (TCGA, https://portal.gdc.cancer.gov/) (Tomczak et al., 2015) and Genotype-Tissue Expression database (GTEx, https://www.gtexportal.org/home/) (GTEx Consortium, 2020). The RNA sequencing data (RNA-Seq) and clinical information of TCGA/GTEx were downloaded from The University Of California Santa Cruz (UCSC) Xena Hub datasets (https://xenabrowser.net/) (Goldman et al., 2015). Meanwhile, all RNA-Seq data normalized with log2(FPKM+1) and merged using the R package “limma”. A total of 263 STS samples and 913 nontumor samples were enrolled in this study for differential analysis and Pearson Correlation analysis. More importantly, 255 STS samples were subjected to subsequent bioinformatics analysis after removing STS samples with incomplete clinical characteristics and non-tumor samples. The detailed clinical characteristics of these patients are shown in Supplementary Table S1.

### Identification of NRlncRNAs

The necroptosis-related genes (NRGs) enrolled in the present study were obtained from the previous literature (Zhao Z. et al., 2021). The detailed information of 67 NRGs was presented in Supplementary Table S2. Then, the Pearson correlation coefficient was calculated based on NRGs and lncRNA expression profiles to identify NRlncRNA (|R^2^| > 0.4 and *p* < 0.001) (Wu et al., 2021).

### Differential Expression Analysis

The “limma” package was used to explore the differentially expressed NRlncRNAs between STS and normal tissue. The NRlncRNA with FDR<0.05 and |log2FC| ≥ 1 was considered as a significant differentially expressed NRlncRNA (Tu et al., 2020). Also, the R package “ggplot2” and “pheatmap” were used to produce volcano plots and heatmaps. To demonstrate the correlation between the differentially expressed NRlncRNAs and their corresponding NRGs, we visualized the co-expression network using Cytoscape v.3.7.0 software (Shannon et al., 2003).

### Construction of NRlncRNA Signature and Its Validation

The STS patients were randomly categorized into a training set (128 STS samples) and a validation set (127 STS samples) to comprehensively explore and verify the prognostic role of NRlncRNA in STS. First, the univariate Cox regression analysis based on the R package ‘survival’ was performed to identify potential prognostic differentially expressed NRlncRNAs. NRlncRNA with *p* < 0.05 was considered a predictive value for overall survival (OS) of STS. Subsequently, NRlncRNAs with prognostic values were further screened by least absolute shrinkage and selection operator (LASSO) regression analysis with choosing the optimal penalty parameter λ correlated with the minimum 10-fold cross-validation. Next, the multivariate Cox regression analysis was conducted to establish and optimize the NRlncRNAs prognosis signature for STS. The risk scores of each patient in this signature are calculated by the following formula: $Risk\;score=\overset{n}{\underset{\text{i}=1}{\sum }}Coef{\left(i\right)}^{\text{∗}}X\left(i\right)$ , where Coef 1) represents the coefficient obtained by multivariate Cox regression, and X 1) represents the expression value of each NRlncRNA.

The Kaplan-Meier (K-M) survival analysis was used to compare the survival difference between the two distinct risk groups in each set (training, validation, and the entire set) using the R package “survminer”. Then, the predictive accuracy of the NRlncRNAs prognostic signature was assessed by the area under the curve (AUC) of the receiver operating characteristic curve (ROC), which was generated by the ‘timeROC’ R package. Also, the predictive value of the novel signature was compared with other clinical characteristics by using the ROC curve. For subsequent analysis and validation, the training set, testing set, and all STS patients were stratified into high- and low-risk groups according to the median risk scores in the training set. Ultimately, the same validation method was performed as above.

### Prognostic and Independent Analysis

The subgroup K-M survival analysis based on the different clinical characteristics was conducted to further assess the sensitivity of the novel signature. OS was compared in the high- and low-risk group. Meanwhile, the univariate COX regression analysis was utilized to investigate the correlation between prognosis and age, gender, recurrence, margin status, metastasis, histological type, and risk score. Subsequently, the multivariate COX regression analysis was performed to identify the independent prognostic value of the novel prognostic signature. Hazard ratio (HR), 95% confidence interval (CI), and p-values were separately determined.

### Nomogram and Calibration

To further assess the predictive capacity of the prognostic signature based on NRlncRNA, we developed a nomogram based on risk scores and other clinical features (age and gender) to estimate the 1-, 2-, and 3-years survival rates of STS patients. Moreover, we then plotted the calibration curve of the nomogram to assess the uniformity between the observed and predicted overall survival. The R package “rms” was used to plot the nomogram and calibration curves.

### Gene Set Enrichment Analysis (GSEA)

Recently, the GSEA was wildly utilized to investigate the potential pathway of the prognosis signature in cancer (Subramanian et al., 2005). In our study, we downloaded Kyoto Encyclopedia of Genes and Genomes (KEGG) gene set (c2. cp.kegg.v7.4. symbols.gmt; c5. go.v7.4. symbols.gmt) from Molecular Signatures Database (MSigDB, http://software.broadinstitute.org/gsea/msigdb/). Subsequently, we applied GSEA via the R package “clusterProfiler” to identify the enriched functional status and biological pathways in high- and low-risk groups. Statistical significance was set at *p* < 0.05 and false discovery rate (FDR) q < 0.25.

### Immune Microenvironment and Enrichment Analysis

Estimation of Stromal and Immune cells In Malignant Tumor tissues using Expression (ESTIMATE) was wildly used to evaluate the presence of stromal and immune cells in the tumor microenvironment based on the expression level of specific genes (Yoshihara et al., 2013). The present study measured the immune infiltration and tumour microenvironment scores of STS patients by ESTIMATE. In addition, differential analysis, correlation analysis, and subgroup survival analysis were applied to reveal the correlation between the novel NRlncRNA signature and the tumor microenvironment in STS.

The single-sample gene set enrichment (ssGSEA) algorithm was conducted based on the specific marker gene expression level of immune cells extracted from the previous study (Bindea et al., 2013). We utilized ssGSEA to calculate the enrichment fraction of various immune cells in STS patients. Also, the differences in immune cell infiltration and immune function between the two-risk group were compared by the Wilcox test.

The CIBERSORT algorithm is a gene expression-based deconvolution algorithm developed by Newman et al., in 2015 to assess immune cell composition (Newman et al., 2015). With this algorithm, we evaluated the infiltration of 22 immune cells in STS patients. Subsequently, the correlation analysis and subgroup survival analysis were performed to explore the impact of immune cell infiltration on the survival prognosis of STS.

### Exploration of the Sensitivity of Chemotherapeutic Agents

To predict the sensitivity of STS with different risk scores to chemotherapeutic agents, we used the R package “pRRophetic” to perform ridge regression to calculate the half-maximal inhibitory concentration (IC50) of STS patients in different risk groups. The IC50 represents a substance’s effectiveness in inhibiting specific biological or biochemical processes. Next, the Wilcoxon signed-rank test was applied to compare the differences in IC50 between the high- and low-risk groups. Twelve anti-tumor drugs such as Axitinib, Bleomycin, Cisplatin, and Doxorubicin were analyzed as candidate agents.

### Cell Culture

The human skin fibroblast cell line (HSF) was purchased from Fenghui Biotechnology Co., Ltd. (Hunan, China). The human synovial sarcoma cell line (SW982) was provided by the American Type Culture Collection (ATCC). The human liposarcoma cell line (SW872) was obtained from Procell Life Science&Technology Co., Ltd. (Hubei, China). All the cell lines were cultured in Dulbecco’s modified Eagle’s medium (Gibco, United States), with 10% fetal bovine serum (Gibco, United States) and 1% penicillin-streptomycin solution (NCM Biotech, China), and the cells were cultured in a humidified atmosphere containing 5% CO2 at 37°C.

### RT-qPCR

According to the manufacturer’s protocol (Qi et al., 2021), the total RNA was extracted with RNA Express Total RNA Kit (M050, NCM Biotech, China). Then, RNA was reverse transcribed using the Revert Aid First Strand cDNA Synthesis Kit (K1622, Thermo Scientific, United States). Finally, Quantitative PCR was performed with Hieff qPCR SYBR Green Master Mix (High Rox Plus) (11203ES, YEASEN Biotech Co., Ltd., China). The reaction conditions were as follows: 95°C for 5 min, followed by 40 cycles of 95°C for 10 s and 60°C for 30 s. GAPDH was selected as the internal reference gene, and the sequences of all primers were given in Supplementary Table S3. The relative expression of lncRNA was determined using the relative quantification method (2^−ΔΔCT^).

### Statistical Analysis

All statistical analyses in the present study were carried out using software R version 4.0.5. Spearman correlation analysis was performed to identify correlation. The survival curves were mapped by K-M survival analysis. Differences between different groups were assessed using independent t-tests or one-way analysis of variance (ANOVA). The univariate and multivariate analyses were performed by Cox proportional hazards model. The PCA analysis was utilized by the R package “scatterplot3d”. If not specified above, the threshold for statistical signiﬁcance was set at *p* < 0.05.

## Results

### Differentially Expressed NRlncRNAs

**FIGURE 1:**
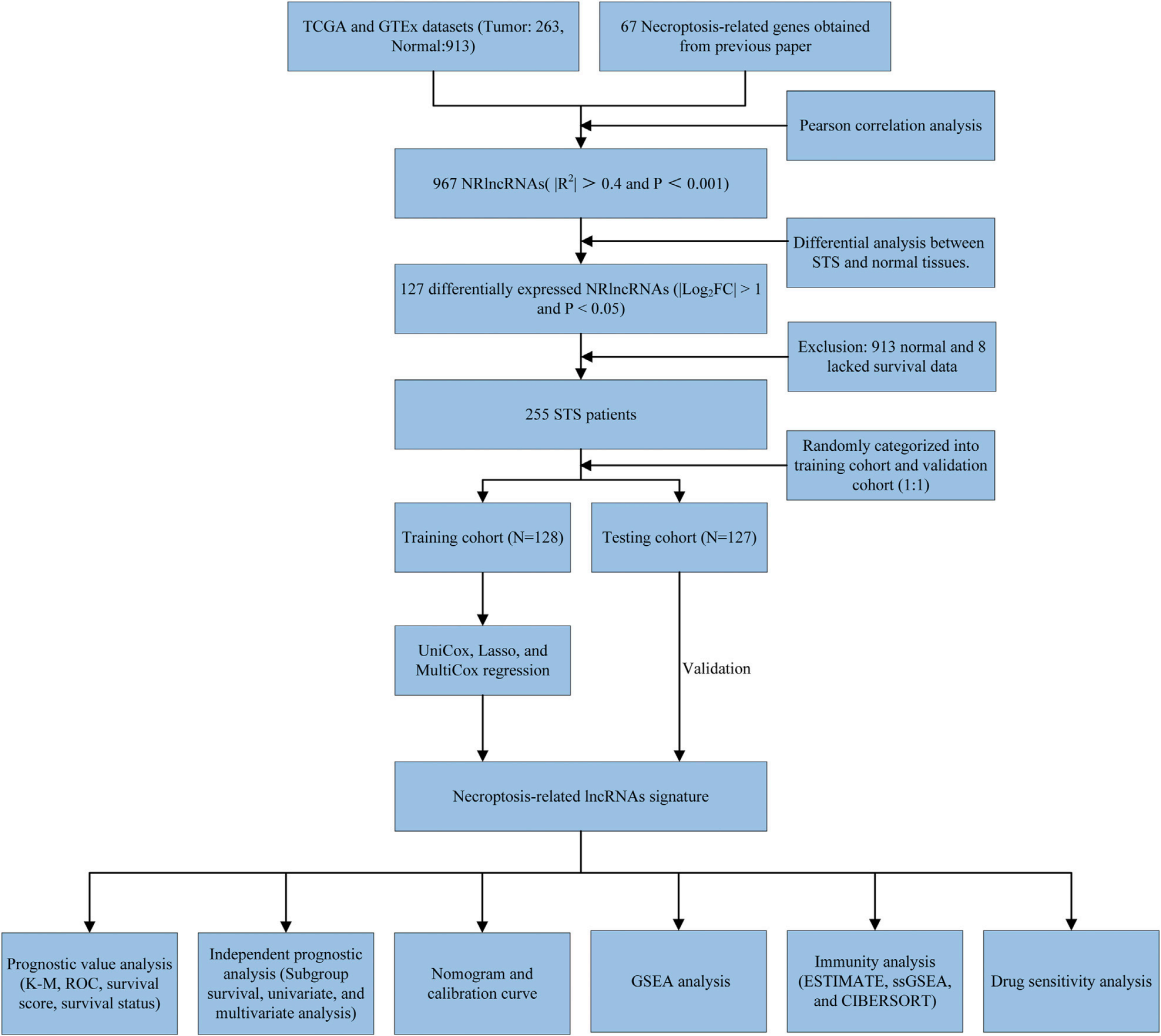
The flow diagram of the present study.

**FIGURE 2:**
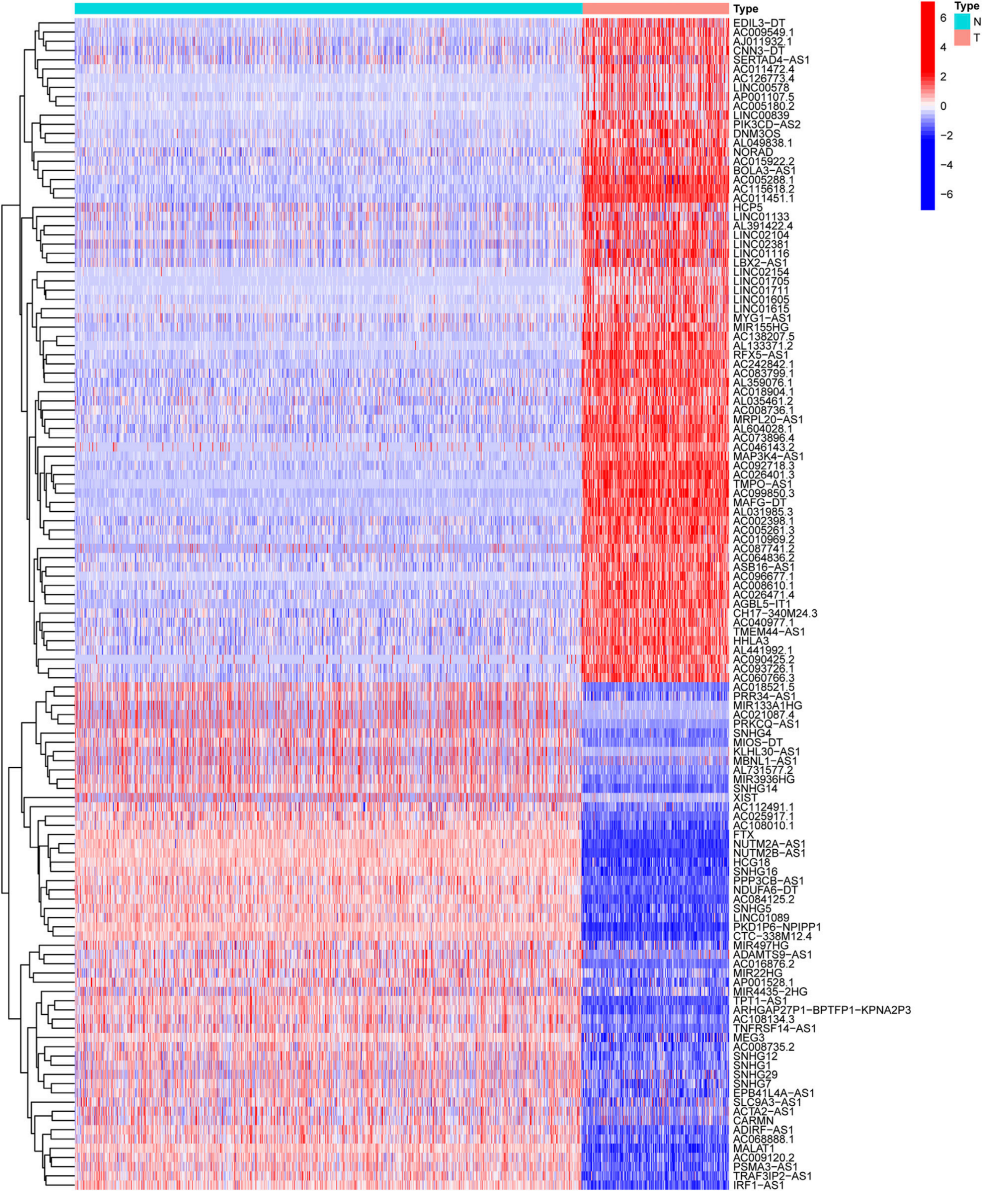
The heatmap of differentially expressed NRlncRNAs.

The workflow of the study is outlined in Figure 1. After normalizing and merging STS tissue and normal tissue expression data, the Pearson correlation analysis was performed between lncRNAs and these 67 NRGs to identify NRlncRNA. In total, 967 NRlncRNAs were defined according to the inclusion parameters (|R^2^| > 0.4 and *p* < 0.001) (Supplementary Table S4). Then, we identified 127 significantly differently expressed NRlncRNAs in STS samples between tumor and normal tissue (FDR<0.05 and |log2FC| ≥ 1). Figure 2 and Supplementary Figure S1 presented the heatmap and volcano plot of these significantly differentially expressed NRlncRNAs, of which 72 were elevated and 55 were downregulated. The detailed information of these 127 NRlncRNAs is listed in Supplementary Table S5. Meanwhile, we observed a significant co-expression association between these NRlncRNAs and NRGs in Supplementary Figure S2.

### Identification and Validation of NRlncRNAs Prognostic Signature

**FIGURE 3:**
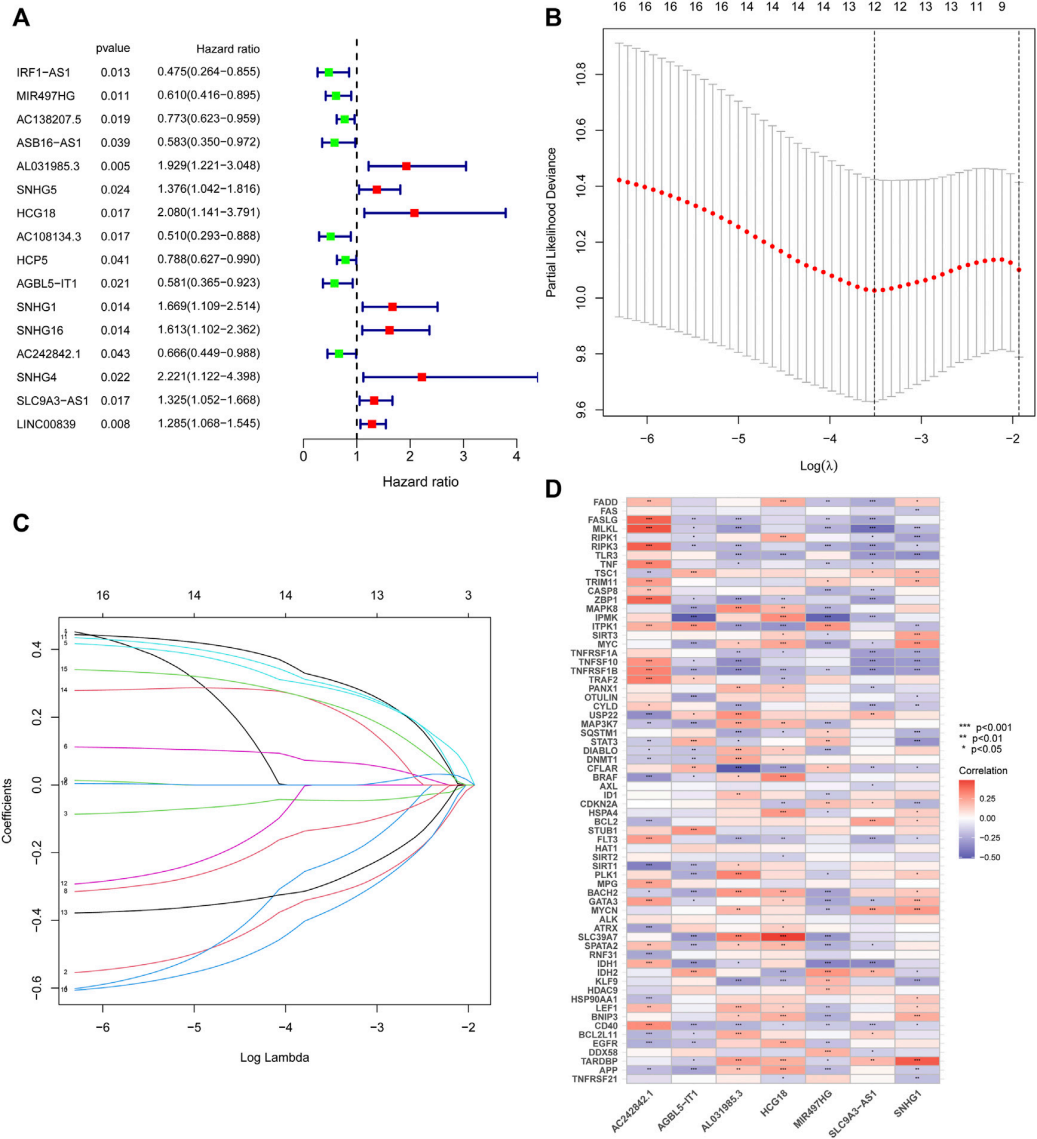
Construction of the NRlncRNAs prognostic signature in STS. **(A)**. Univariate cox regression analysis of STS for each NRlncRNAs. **(B)**. Cross-validation for tuning the parameter selection in the LASSO regression. **(C)**. LASSO regression of these NRlncRNAs. **(D)**. The co-expression relationship of these 7 NRlncRNAs and NRGs.

**FIGURE 4:**
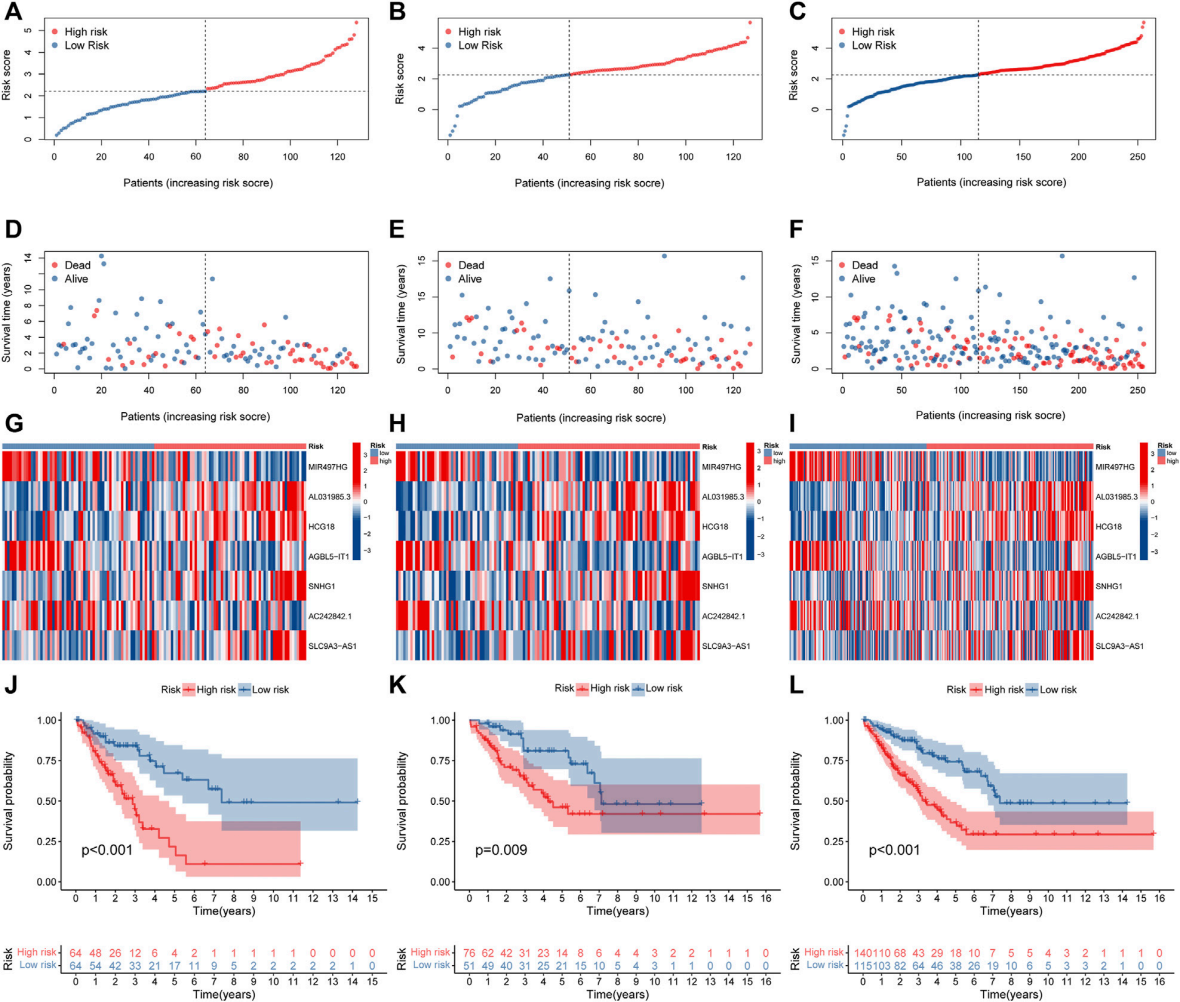
Prognosis value of the novel NRlncRNAs signature in the train, test, and entire sets. **(A–C)**. The distribution of patient risk ratings in the train, test, and entire sets, respectively. **(D–F)**. The plot of risk survival status in the train, test, and entire sets, respectively. **(G–I)**. The heat map of 7 lncRNAs expression in the train, test, and entire sets, respectively. **(J–L)**. The Kaplan–Meier survival of STS patients between low- and high-risk groups in the train, test, and entire sets, respectively.

The above 127 NRlncRNAs were used for further analysis. First, a total of 16 NRlncRNAs significantly interrelated with the prognosis of STS were screened by univariate COX regression analysis (Figure 3A). Subsequently, 12 NRlncRNAs remained after correction by LASSO regression analysis (Figures 3B,C). Next, the above NRlncRNAs correlated with OS were subjected to multivariate COX regression analysis for signature optimization and construction. As a result, a novel prognostic signature composed of 7 NRlncRNAs was identified, including MIR497HG, AL031985.3, HCG18, AGBL5-IT1, SNHG1, AC242842.1, and SLC9A3-AS1. Figure 3D shows the co-expression relationship between these seven signature lncRNAs and NRGs, and we can observe that most NRGs were significantly correlated with the seven lncRNAs. Accordingly, the formula of risk scores is follow: Risk score = (-0.512253526600089) ^∗^ MIR497HG + 0.424292247918774 ^∗^ AL031985.3 + 0.556753388266948 ^∗^ HCG18 + (-0.588420976123074) ^∗^ AGBL5-IT1 + 0.523706712328759 ^∗^ SNHG1 + (-0.472715571972488) ^∗^ AC242842.1 + 0.320232600341058 ^∗^ SLC9A3-AS1. With the risk score formula, we compared the distribution of risk scores, survival status, and expression level of NRlncRNAs between the low-risk and high-risk groups in the training, testing, and entire groups. As shown in Figures 4A–I, we found that the dead patients were mainly distributed in the high-risk group. Furthermore, we applied K-M survival analysis between the two distinct groups to investigate the association between the novel risk signature and the clinical prognosis of STS patients. As presented in Figures 4J–L, the survival rate of STS patients in the high-risk group was significantly lower than that in the low-risk group, neither in terms of training, testing group, nor the entire group. Taken together, these results demonstrated a poorer prognosis in STS with high-risk scores.

### Assessment of the Novel Risk Signature

**FIGURE 5:**
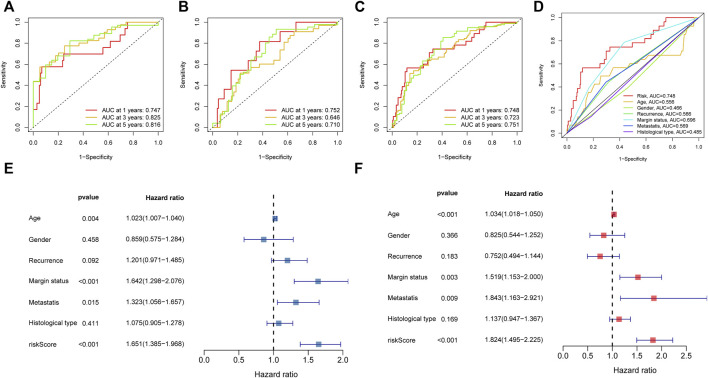
The prognosis value of the novel NRlncRNAs signature. **(A–C)**. The 1-, 3-, and 5-years ROC curves of the train, test, and entire sets, respectively. **(D)**. The ROC curves of risk score and clinical characteristics. **(E)**. The result of univariate Cox regression analysis. **(F)**. The result of multiple Cox regression analysis.

To further assess the sensitivity and specificity of the signature in terms of prediction, we plotted ROC curves. According to the AUC, we knew that the model has superior prognostic value either in the training, test, or entire groups. The corresponding result of ROC was illustrated in Figures 5A–C. In parallel, the AUC of risk score was also higher than other clinical characteristics, such as age and gender (Figure 5D). In addition, the PCA analysis revealed that the novel signature based on signature NRlncRNAs could better separate STS patients with different risks (Supplementary Figure S3). Hence, the above results indicated that the novel NRlncRNA prognostic signature has outstanding potential for predicting prognosis in the STS cohort.

### Independent Prognostic Value of the Novel Prognostic Signature

We then carried out subgroup survival analysis and Cox regression analysis to investigate whether the NRlncRNAs signature was independent of other clinical features. The STS patients in the high-risk group had poorer OS regardless of the clinical subgroups (Age, Gender, Histological type, Margin status, and Recurrence subgroup), suggesting that the prognosis prediction of our novel signature was robust (Supplementary Figure S4). Moreover, the univariate Cox regression analysis result demonstrated that the risk score was significantly associated with the OS of STS (Figure 5E). Simultaneously, the multivariate Cox regression also showed a significant correlation between the risk score and the prognosis of STS (Figure 5F). Collectively, the risk score based on the novel NRlnRNAs signature was an independent prognostic predictor for STS.

### Establishment of the Nomogram

**FIGURE 6:**
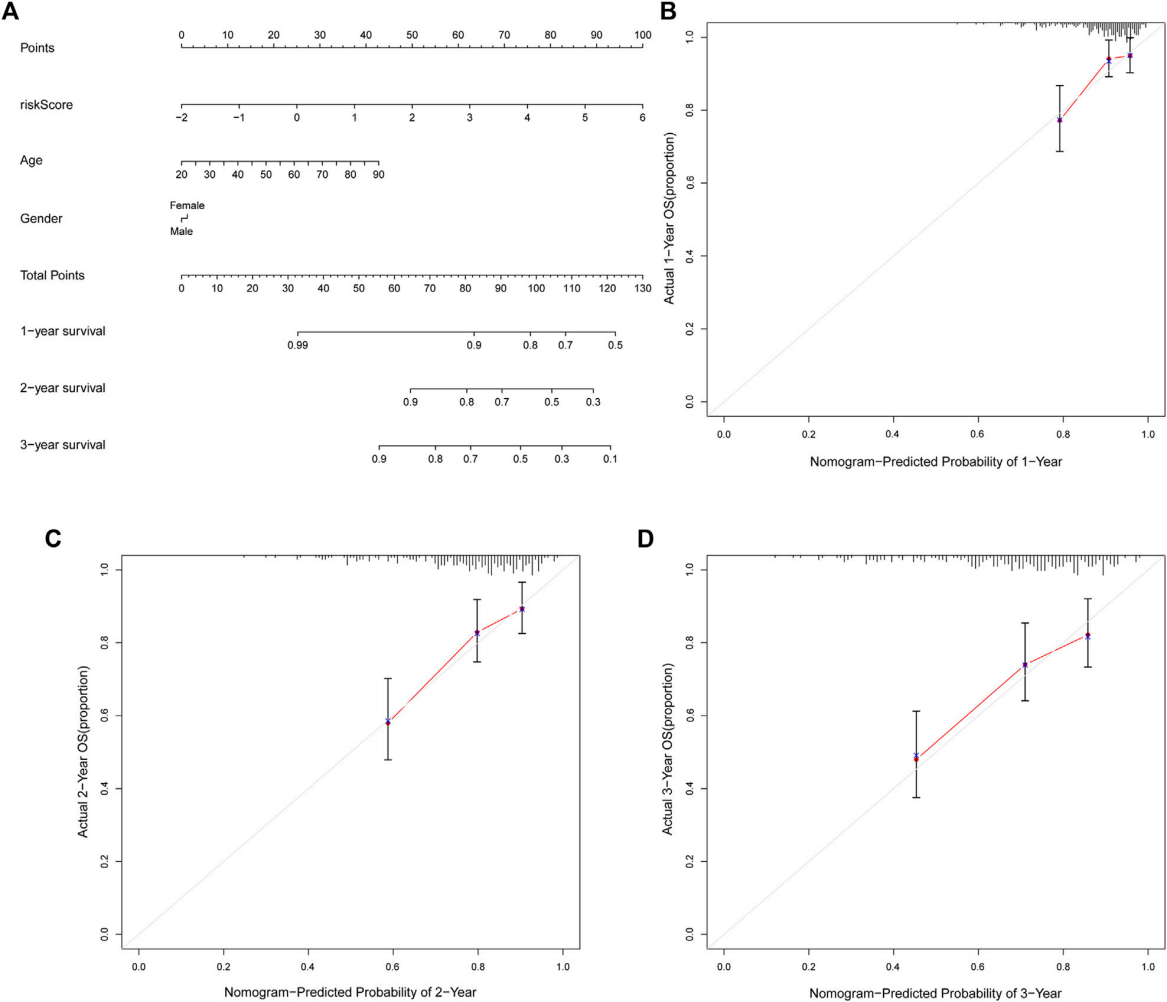
The nomogram can predict the prognosis probability in STS. **(A)**. The nomogram consisting of NRlnRNA signature risk score, age, and gender, which could help predict STS survival rate within 1-, 2-, and 3- years. **(B–D)**. Calibration curve for the 1-, 2-, and 3-years predicted survival nomogram.

The nomogram can assist clinical individualized prognosis prediction and guide treatment strategy (Huang et al., 2019). To facilitate the clinical utility of the novel NRlncRNA signature in predicting the survival rate of STS patients, we constructed a nomogram based on clinical characteristics and the novel NRlncRNA signature to predict the probability of 1-, 2-, and 3-years overall survival of OS. The risk score, age, and gender were included in the nomogram (Figure 6A). Each patient was evaluated by clinically relevant variables in the nomogram and assigned a score, and then each patient could get a total score for predicting their survival rate within 1-, 2-, and 3- years. Additionally, the calibration curve was drawn to evaluate the predictive capacity of the nomogram. As shown in Figures 6B–D, the predicted lines overlapped well with the diagonal lines, suggesting that the nomogram-predicted overall survival within 1-, 2-, and 3- years was consistent with actually observed overall survival of STS. As described, these results further confirmed the stability of the prognostic signature.

### Functional Enrichment Analysis

**FIGURE 7:**
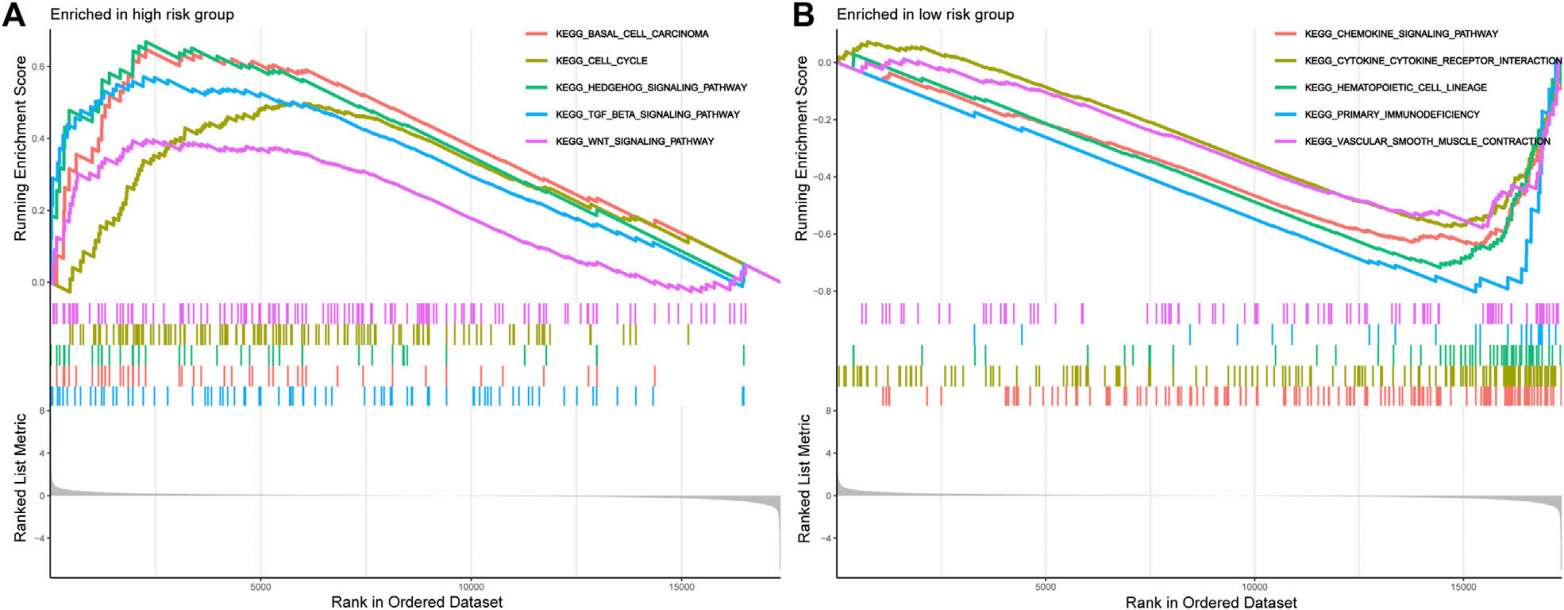
The GSEA analysis results between the high-risk group and low-risk group. **(A)**. GSEA of the top 5 pathways significantly enriched in the high-risk group. **(B)**. GSEA of the top 5 pathways significantly enriched in the low-risk group.

Next, the GSEA has successfully revealed the molecular function difference between the high- and low-risk groups. As illustrated in Figure 7A, the high-risk STS patients are mainly enriched in Basal cell carcinoma, Cell cycle, Hedgehog signaling pathway, TGF beta signaling pathway, and Wnt signaling pathway. Instead, the low-risk group is mainly involved in immune-related pathways, including Chemokine signaling pathway, Cytokine-cytokine receptor interaction, Primary immunodeficiency, Toll-like receptor signaling pathway, Natural killer cell mediated cytotoxicity, Antigen processing and presentation, and NOD-like receptor signaling pathway (Figure 7B and Supplementary Table S6). Consequently, these results hinted that these signal pathways might be the potential mechanisms that result in the poor clinical prognosis of STS.

### The NRlncRNAs Are Closely Associated With Tumor Immunity

**FIGURE 8:**
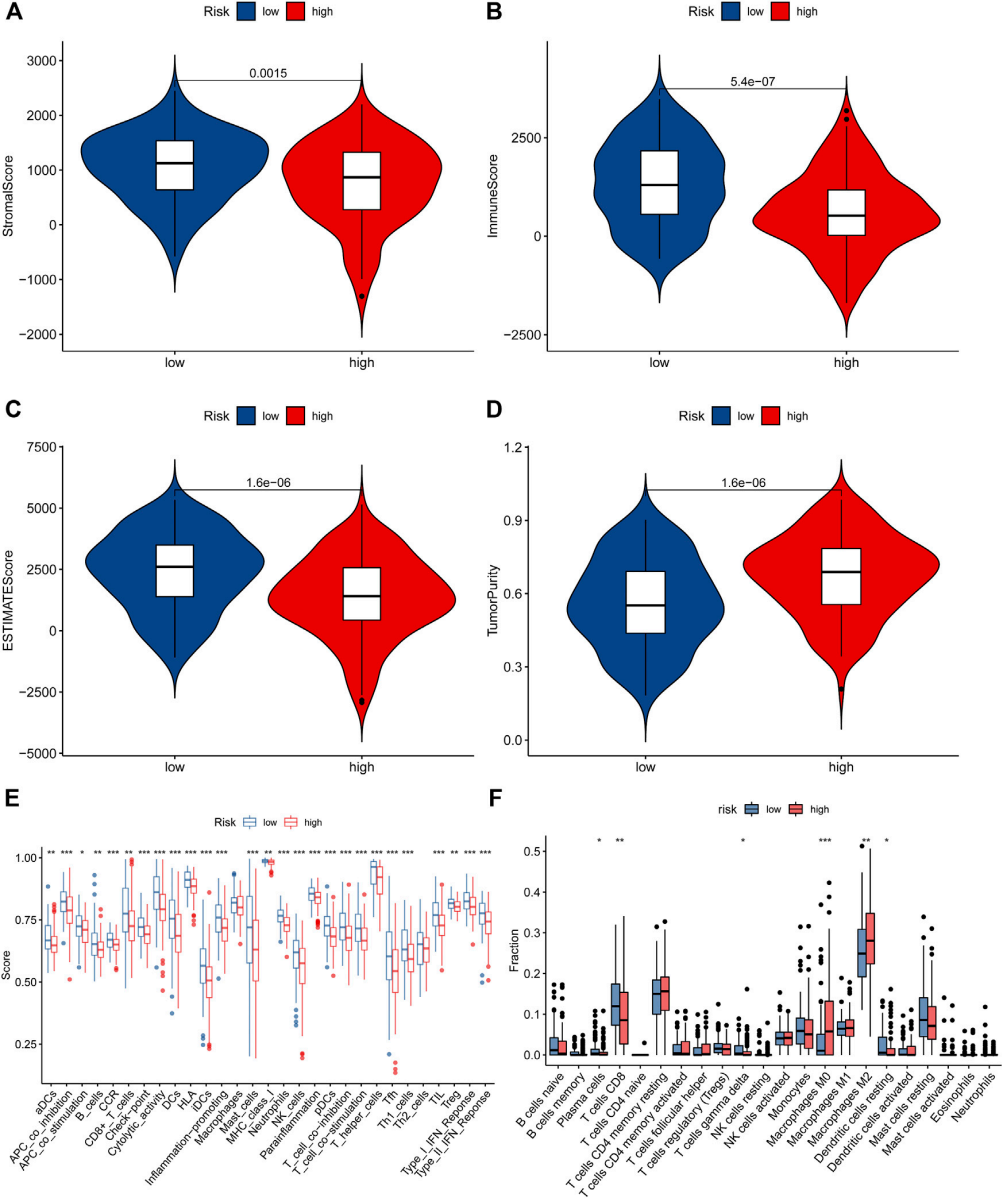
The immune status in STS patients. **(A–D)**. The difference in stomal score, immune score, ESTIMATE score, and tumor purity score between low-risk and high-risk groups. **(E)**. The ssGSEA algorithm result. **(F)**. The violin plot showed the different proportions of tumor-infiltrating cells between the high-risk and low-risk groups.

To reveal the correlation between the prognostic signature based on NRlncRNAs and the tumor immune microenvironment, we evaluated the immune score, immune function, and immune cell infiltration in STS by ESTIMATE, ssGSEA, and CIBERSORT algorithm. First, the result of ESTIMATE exhibited that the STS patients in the low-risk group had higher immune, stromal and ESTIMATE scores and a lower tumor purity score than that of the high-risk group (Figures 8A–D). Additionally, there was a significant negative correlation between risk scores and immune, stromal and ESTIMATE scores, while the tumor purity score was positively related to the risk score (Supplementary Figure S5). In parallel, the K-M survival analysis demonstrated that the STS patients with a higher stromal, immune, and ESTIMATE score had a better survival rate, while a higher tumor purity score was related to a poor prognosis (Supplementary Figure S6). Additionally, we compared the enrichment fraction of immune cells and the activity of immune-related pathways between the two risk groups. As presented in Figure 8E and Supplementary Figure S6, the most immune cells and immune-related pathways in the low-risk group were more active than those in the high-risk group, except for Macrophages, Th2_cells. It is compatible with previous ESTIMATE analysis, indicating that STS patients had more excellent immune status in the low-risk group. Subsequently, we further applied the CIBERSORT algorithm to examine the proportions of the immune landscape in STS. As observed in Figure 8F, there was a significant difference in the proportion of Plasma cells, T cell CD8, T cells gamma delta, Macrophages M0, Macrophages M2, and Dendritic cells resting. Equally, the abundance of these immune cells infiltrated was significantly correlated with risk scores and the clinical prognosis of STS (Supplementary Figure S7). Finally, we also compared the expression of immune checkpoints in different risk groups. The results demonstrated that the expression of most immune checkpoints was significantly elevated in the low-risk group compared with the high-risk group (Supplementary Figure S8). Overview, these findings implied that the novel signature is associated with the immune microenvironment, and the poor prognosis of high-risk STS patients may have resulted from poor immune status.

### The Relationship Between the Novel Signature and Common Chemotherapeutics

**FIGURE 9:**
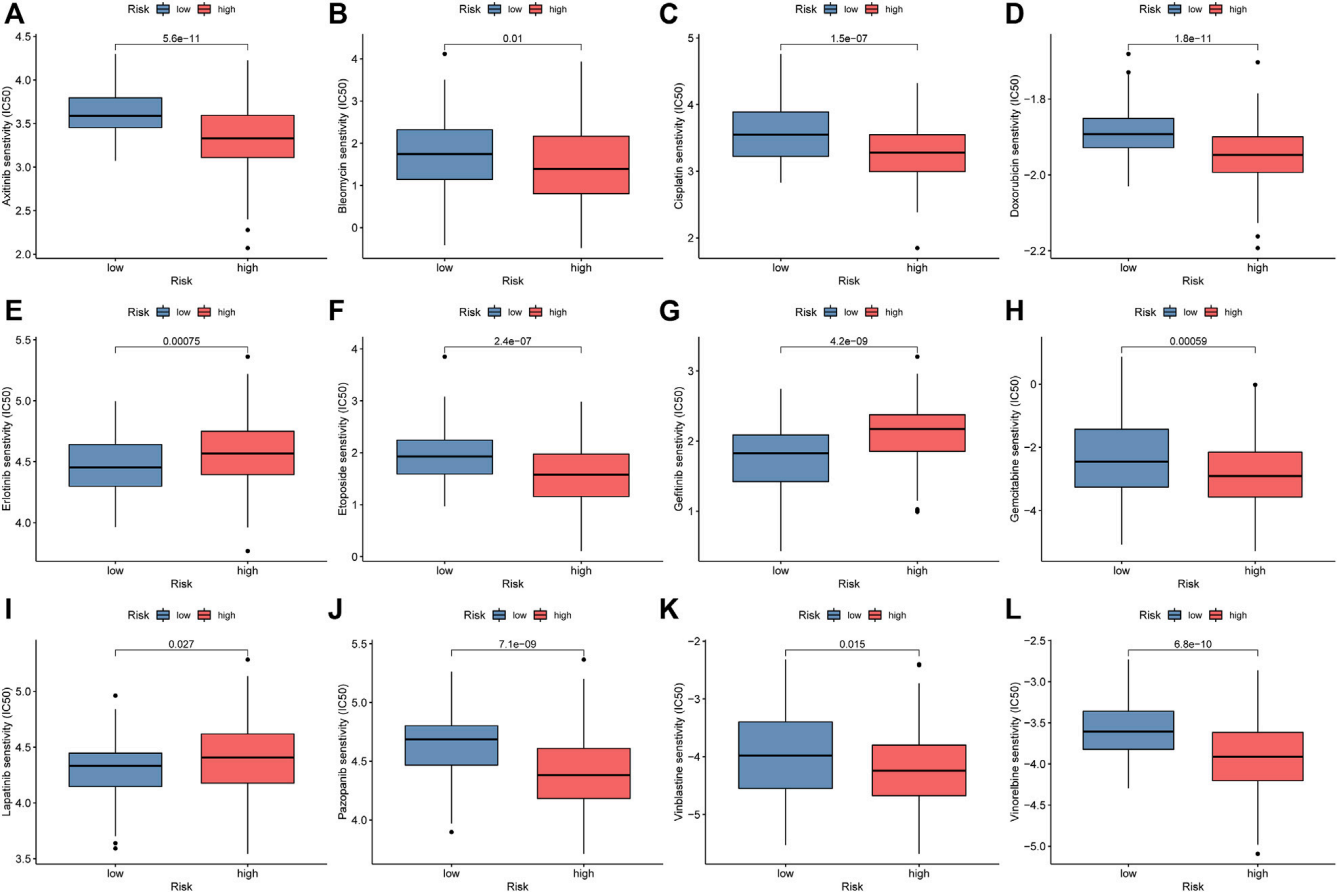
Comparison of IC50 of anti-tumor drugs between the low-risk and high-risk groups. **(A)**. Axitinib, **(B)**. Bleomycin, **(C)**. Cisplatin, **(D)**. Doxorubicin, **(E)**. Erlotinib, **(F)**. Etoposide, **(G)**. Gefitinib, **(H)**. Gemcitabine, **(I)**. Lapatinib, **(J)**. Pazopanib, **(K)**. Vinblastine, **(L)**. Vinorelbine.

To explore the value of the NRlncRNA signature for the clinical treatment of STS patients, we further compared the association between risk scores and the efficacy of commonly used anti-tumor agents. The results demonstrated that the STS patients with higher risk scores were relevant to better respond to most anti-tumor drugs, such as Axitinib, Bleomycin, Cisplatin, Doxorubicin, etc. On the contrary, the STS patients with lower risk scores had excellent sensitivity for Erlotinib, Gefitinib, and Lapatinib (Figure 9). Given the above, we believe that the novel signature composed by NRlncRNAs has promising predictive value for chemosensitivity.

### Verification of the NRlncRNAs in STS Cell

**FIGURE 10:**
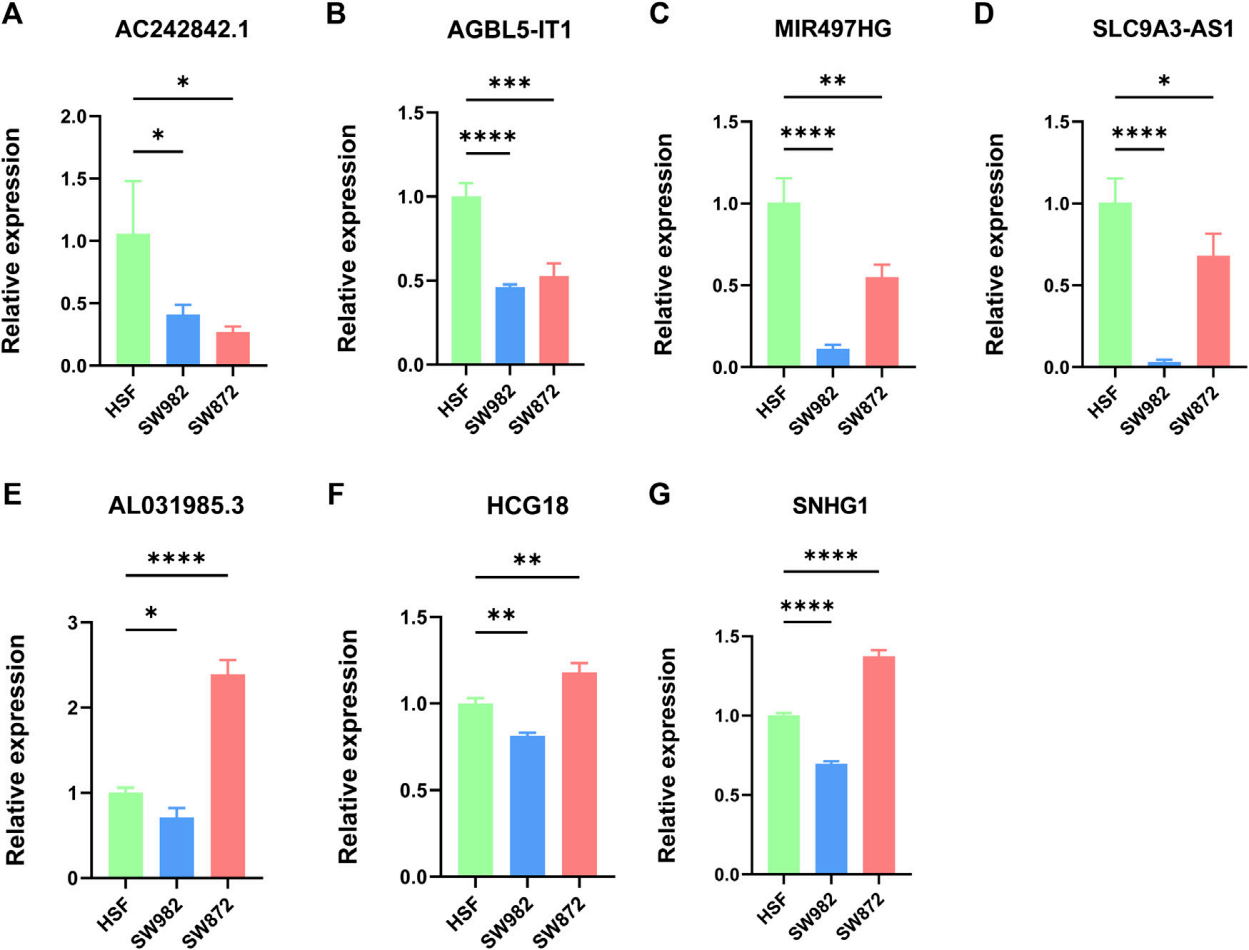
**(A–G)**. Evaluation of the expression of these seven signature NRlncRNAs in STS cell lines. **(A)**. AC242842.1. **(B)**. AGBL5-IT1. **(C)**. MIR497HG. **(D)**. SLC9A3-AS1. **(E)**. AL031985.3. **(F)**. HCG18. **(G)**. SNHG1. *p < 0.05, **p < 0.01, ***p < 0.001, ****p < 0.0001.

We further explore the expression level of these signature NRlncRNAs using STS cell lines. The result indicated that AC242842.1, AGBL5-IT1, MIR497HG and SLC9A3-AS1 were statistically downregulated in SW982 and SW872 cell lines (Figures 10A–D). The AL031985.3, HCG18, and SNHG1 were diminished in the SW982 cell line while elevated in the SW872 cell line (Figures 10E–G). Hence, these results demonstrated a significant difference in these signature NRlncRNAs expression between STS and normal tissue, which implied the importance of these signature NRlncRNAs in STS and provided potential diagnosis and therapy biomarker for STS.

## Discussion

STS is a group of rare and aggressive malignancies originating from mesenchymal tissue, including various pathological subtypes (Dotan et al., 2006). A multidisciplinary manner composed of surgery, radiotherapy, and chemotherapy is the routine treatment for STS (Zhao L. et al., 2021). Generally, the 5-years survival rate of patients with localized STS can reach 55.5–56.5%, but these traditional treatments have unsatisfactory curative effects on patients with postoperative metastasis and recurrence (Hu et al., 2020). Therefore, searching for precise biomarkers for STS patients has vital significance for guiding the clinical treatment and prognosis prediction of STS patients. An increasing body of evidence demonstrated that necroptosis acts a vital role in carcinogenesis, which may be a potential therapeutic target for STS. Similarly, it has been proved that lncRNA could serve as an effective prognostic biomarker for STS. As an example, Lin et al. revealed that the pyroptosis-related lncRNAs could accurately predict the clinical prognosis and immune microenvironment of STS (Lin et al., 2021). More importantly, previous studies have confirmed that the NRlncRNA signature also can be used to predict the prognosis of tumors. For instance, the NRlncRNAs have vital functions in Colon Cancer, and the signature composed of NRlncRNAs can predict the prognosis of Colon Cancer (CC) and be used to customize CC treatment in the future (Liu et al., 2022). However, the relationship between NRlncRNAs and STS is still unknown. Therefore, our study firstly constructs a novel prognostic signature based on NRlncRNAs and comprehensively investigates the prognostic role of NRlncRNAs in STS.

The present study identified 127 significantly differently expressed NRlncRNAs through co-expression analysis and differential analysis based on 67 NRGs extracted from the previous paper. Next, a novel prognosis signature consisting of MIR497HG, AL031985.3, HCG18, AGBL5-IT1, SNHG1, AC242842.1, and SLC9A3-AS1 was established successfully by the COX regression analysis and LASSO regression analysis. The K-M survival analysis exhibited that the model was significantly associated with the OS of STS. The ROC curve further reflected the superior predictive ability of the signature. Consistently, these results were verified by the testing and entire sets. Ultimately, the univariate, multivariate Cox regression analysis and subgroup survival analysis also confirmed that the risk score of the prognostic signature could be used as an independent prognostic factor for STS. Hence, we believe that the novel NRlncRNAs signature has relatively stable prediction performance and general applicability.

Accurate prognostic assessment is crucial for selecting suitable treatment options. A nomogram is a prediction tool based on a statistical regression model which provides a numerical prediction of a clinical survival for the individual patient via a simple calculation in clinical practice. In the present study, we successfully established a nomogram containing an NRlncRNA risk score that could predict the OS of STS. In addition, the calibration curve displays large similarities between the predicted OS and actual OS, indicating the steady predictive performance of the nomogram.

The GSEA was an effective method to reveal the essential biological processes in tumorigenesis, which was widely used and highly reliable. Therefore, we performed GSEA to uncover underlying molecular mechanisms that contribute to the poor prognosis of STS patients. According to the results, the patients in the high-risk group were significantly enriched in multiple tumor-related pathways, which have been found related to the occurrence and development of STS in previous studies. For instance, Tan et al. proved that PLA2G10 facilitates the cell-cycle progression of soft tissue leiomyosarcoma (STLMS) cells via upregulating the expression of cyclin E1/CDK2, which was a crucial oncogenic property in STLMS(Tan et al., 2020). Likewise, the activation of the TGF-beta signalling pathway was shown to promote the migration and invasion of osteosarcoma (Chen et al., 2019). In addition, the Wnt signalling pathway also plays an essential role in regulating osteosarcoma function (Singla et al., 2020). However, the low-risk STS patients were enriched in several immune-related pathways, such as the Toll-like receptor signaling pathway and NOD-like receptor signaling pathway. This phenomenon has also been observed in other studies as well. Previously, Yun et al. demonstrated that the novel ferroptosis-related lncRNAs prognostic signature could regulate the immune pathways in Head and neck squamous cell carcinoma (Tang et al., 2021). Moreover, a great of studies have shown that tumor immunity plays an important role in tumors (Sengupta et al., 2010). Therefore, we speculate that the OS of different risk STS patients may be related to tumor immunity, but the relationship between them needs further evaluation.

Considering the critical role of immune infiltration in the occurrence of STS (Que et al., 2017) and the above GSEA result, we further performed a comprehensive correlation analysis to explore the differences in immune status between the two risk groups. First, the ESTIMATE and ssGSEA results revealed that the risk score of the NRlncRNAs prognostic signature was negatively correlated with the immune score, immune cells, and immune-related functions in STS. Equally, STS patients in high-risk groups had elevated expression of the most checkpoint than those in lower-risk groups. There were similar results in the previous studies. Lin et al. demonstrated that the STS patients with a lower pyroptosis-related risk score had an upregulated expression of several checkpoints (Lin et al., 2021). Surprisingly, the CIBERSORT algorithm indicated that the risk score and clinical prognosis of STS also were significantly associated with the infiltration of some immune cells. Among them, macrophages M0 and macrophages M2 were the only two types of immune cells that could promote the STS progress in the necroptosis-related pathway. Typically, macrophage M0 is the unactivated subtype that can polarise into the M1 or M2 subtypes (Locati et al., 2020). It has been shown that the M1 subtype holds a pro-inflammatory, anti-neoplastic effect while the M2 macrophages with a pro-tumorigenic effect (Anderson et al., 2021). Also, previous studies have revealed that macrophage M0 has opposite effects on STS, which was compatible with our results (Zhu and Hou, 2020; Fan et al., 2021). As described, these results and previous studies suggested the effects of NRlncRNAs on the prognosis of STS via modulating the infiltration of immune cells, and the abundance of macrophages M0 and M2 could be used as a critical biomarker to distinguish the clinical prognosis of STS.

To further apply the novel signature to the clinical use for improving treatment efficacy, the association between the risk scores and the sensitivity of anti-tumor agents was explored in-depth via “pRRophetic”. The IC50 of nine anti-tumor agents in the low-risk group was significantly higher than in the high-risk group. On the contrary, the STS in the high-risk groups presented greater sensitivity to Erlotinib, Gefitinib, and Lapatinib. A similar result was previously reported by Yang et al., who demonstrated that the hepatocellular carcinoma patient with lower angiogenesis-related immune signature risk scores has higher chemosensitivity to sorafenib (Yang et al., 2021). Equally, the anti-tumor effect of these drugs is indubitable. For instance, Erlotinib has anticancer effects on a wide range of malignancies, especially non-small cell lung cancer (Brower and Robins, 2016; Tagliamento et al., 2018). Hence, it can be concluded that the novel signature based on NRlncRNAs has a promising perspective for its potential clinical applicability in STS, such as in the optimization of personalized therapy.

Finally, we preliminarily evaluated the expression of these signature NRlncRNAs in the STS cell line by RT-qPCR. Surprisingly, the result confirmed that these NRlncRNAs expression levels were abnormally expressed in STS, hinting that these lncRNAs may fulfil distinct functional roles in STS. In parallel, updates confirm that these several NRlncRNAs may fulfil critical functional roles in various tumors. For instance, there are significant differential expression levels of SLC9A3-AS1 between lung cancer patients and healthy controls (Bai et al., 2019). Also, the novel lncRNA MIR497HG considered new tumor suppressors and prognostic biomarkers in glioma and significantly inhibited glioma cell proliferation ability and cell cycle progression via targeting CCNE1 and the miR-588/TUSC1 axis (Ji et al., 2021). However, the specific role of these NRlncRNAs in STS needs to be thoroughly studied in the future.

There were still several limitations remained though efforts have been attempted to use many methods for evaluation of our signature. First, we had conducted internal validation by the testing and entire cohort in the novel signature, but it was difficult to perform independent external validation for prognoses. Since the external database may have the biases and limitations when compared with TCGA and GTEx, we could not extract appropriate lncRNA profile as an independent external cohort for validation. This situation is consistent with several other prior studies utilizing a similar approach (Wu et al., 2020; Bu et al., 2021; Gao and Li, 2021; Qu et al., 2022). However, we performed the RT-qPCR to detect the expression of these signature lncRNAs, which may be recognized as an external validation. Given the above analyses and previous investigations, we believe that the novel NRlncRNA prognostic signature was reasonable and acceptable for future clinical tests. Second, most of our results preliminarily revealed that the novel NRlncRNA prognostic signature and the prognosis of STS patients might be relevant to the immune microenvironment. However, its specific functions and regulatory mechanisms of NRlncRNA in the immune microenvironment in STS remained largely unknown and required further exploration. Last, the RT-qPCR results of the expression level trend of AL031985.3, HCG18, and SNHG1 in different STS cell lines were not always consistent. Since STS refers to a group of tumors with high heterogeneity, such as liposarcoma, synovial sarcoma, fibrosarcoma, etc., the validation tests based on these different cell lines may lead to potential inconsistency. Therefore, more clinical datasets are still needed to further verify the values of the novel NRlncRNAs signature in each specific STS in the future.

## Conclusion

In summary, we comprehensively assessed the prediction value of NRlncRNAs, the possible biological mechanisms of the novel NRlncRNAs signature, the association between tumor immune and NRlncRNAs, and predictive therapy potential agents for STS. It can be concluded that the novel signature based on NRlncRNAs may help the clinical survival prediction and personalized treatment of STS patients in the future.

## Data Availability

The datasets generated and analyzed during the current study are available in public databases. This data can be found here: TCGA: https://portal.gdc.cancer.gov/, GTEx: https://www.gtexportal.org/home/, UCSC: https://xenabrowser.net/.

## Abbreviations

AUC, area under the curve; AML, acute myeloid leukemia; ATCC, American Type Culture Collection; CC, Colon Cancer; ESTIMATE, Estimation of Stromal and Immune cells In Malignant Tumor tissues using expression; FDR, false discovery rate; GTEx, Genotype-Tissue Expression database; GSEA, Gene set enrichment analysis; IC50, inhibitory concentration; KEGG, Kyoto Encyclopedia of Genes and Genomes; K-M, Kaplan-Meier; LncRNA, long noncoding RNA; LASSO, least absolute shrinkage and selection operator; MLKL, mixed lineage kinase-like; MSigDB, Molecular Signatures Database; NRlncRNAs, necroptosis-related lncRNAs; NRGs, necroptosis-related genes; OS, overall survival; PCD, programmed cell death; RIPK1, receptor-interacting kinase 1; RIPK3, receptor-interacting kinase 3; RNA-Seq, RNA sequencing; ROC, receiver operating characteristic curve; STS, soft tissue sarcoma; ssGSEA, single-sample gene set enrichment; STLMS, soft tissue leiomyosarcoma; TCGA, The Cancer Genome Atlas; UCSC, The University Of California Santa Cruz.
