# Supplementary material for: A Necroptosis-Related lncRNA Signature Predicts Prognosis and Indicates the Immune Microenvironment in Soft Tissue Sarcomas
Source: Front Genet. 2022 Jun 20;13:899545. doi: 10.3389/fgene.2022.899545 (PMC9251335; doi:10.3389/fgene.2022.899545)
Supplement: Supplementary file 1 [file DataSheet1.zip › Supplementary Figure.docx]

**Supplementary Figures**

**
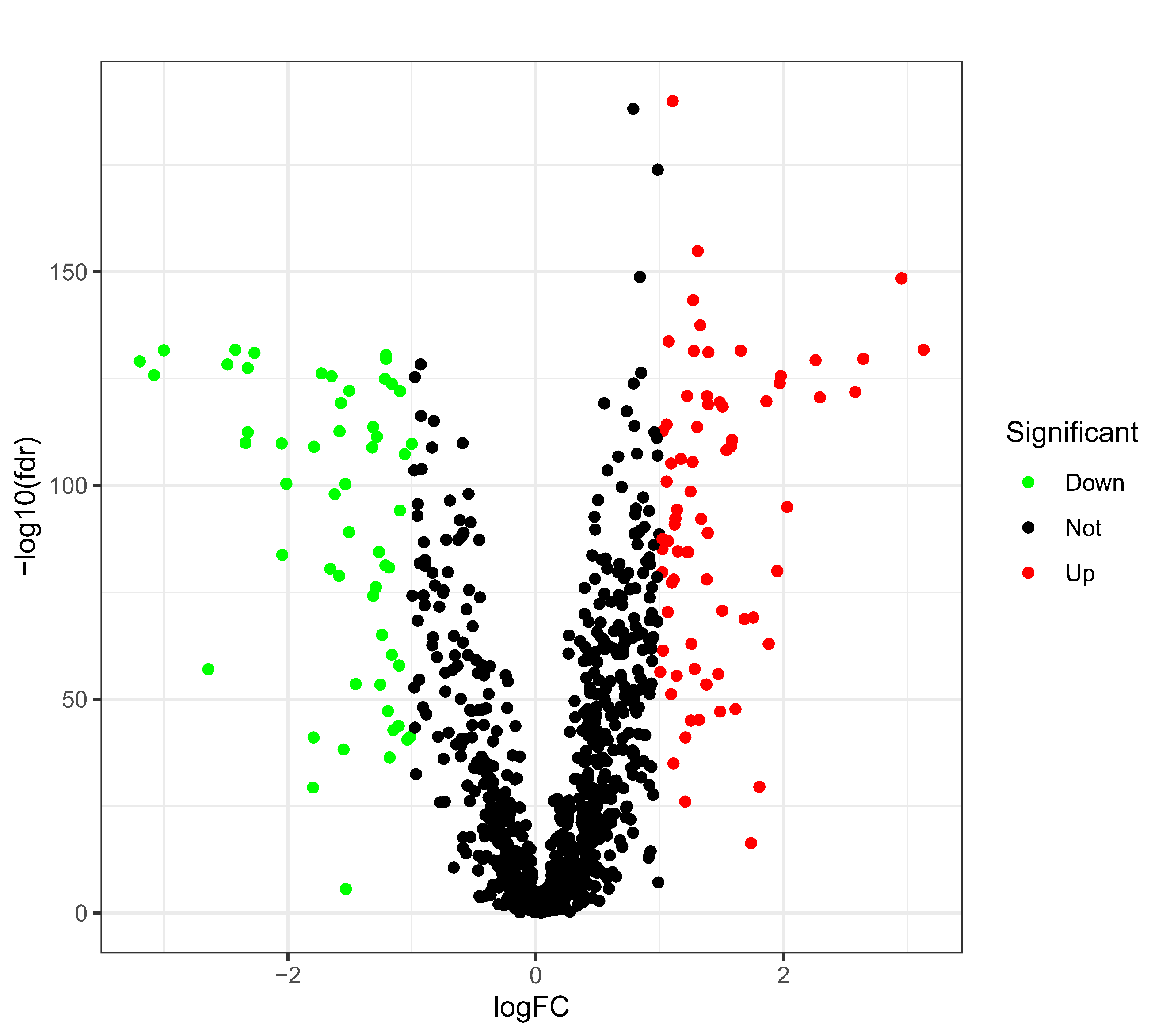
**

**Figure S1.** The volcano of NRlncRNAs.


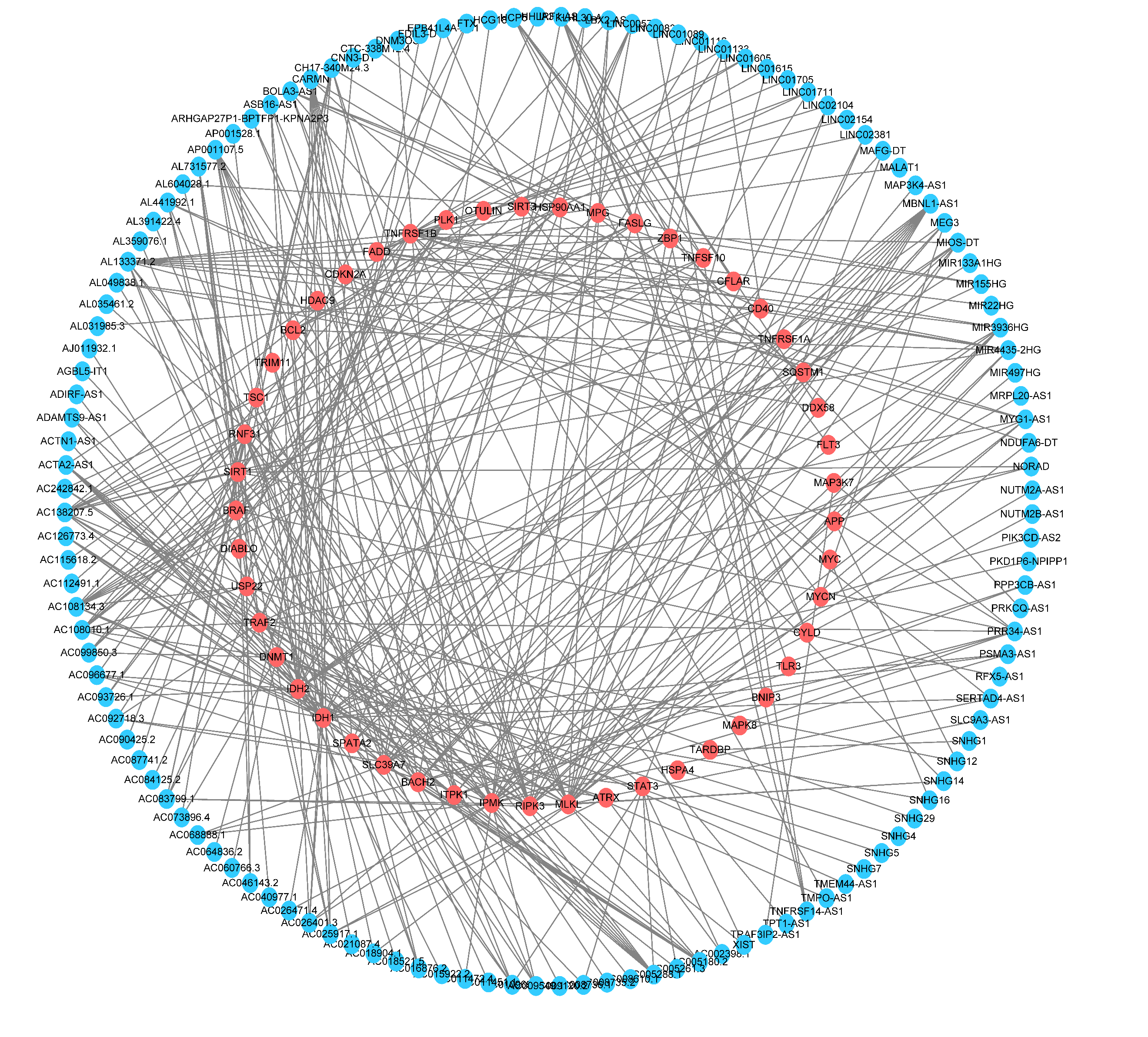


**Figure S2.** The co-expression network between these NRlncRNAs and NRGs.

**
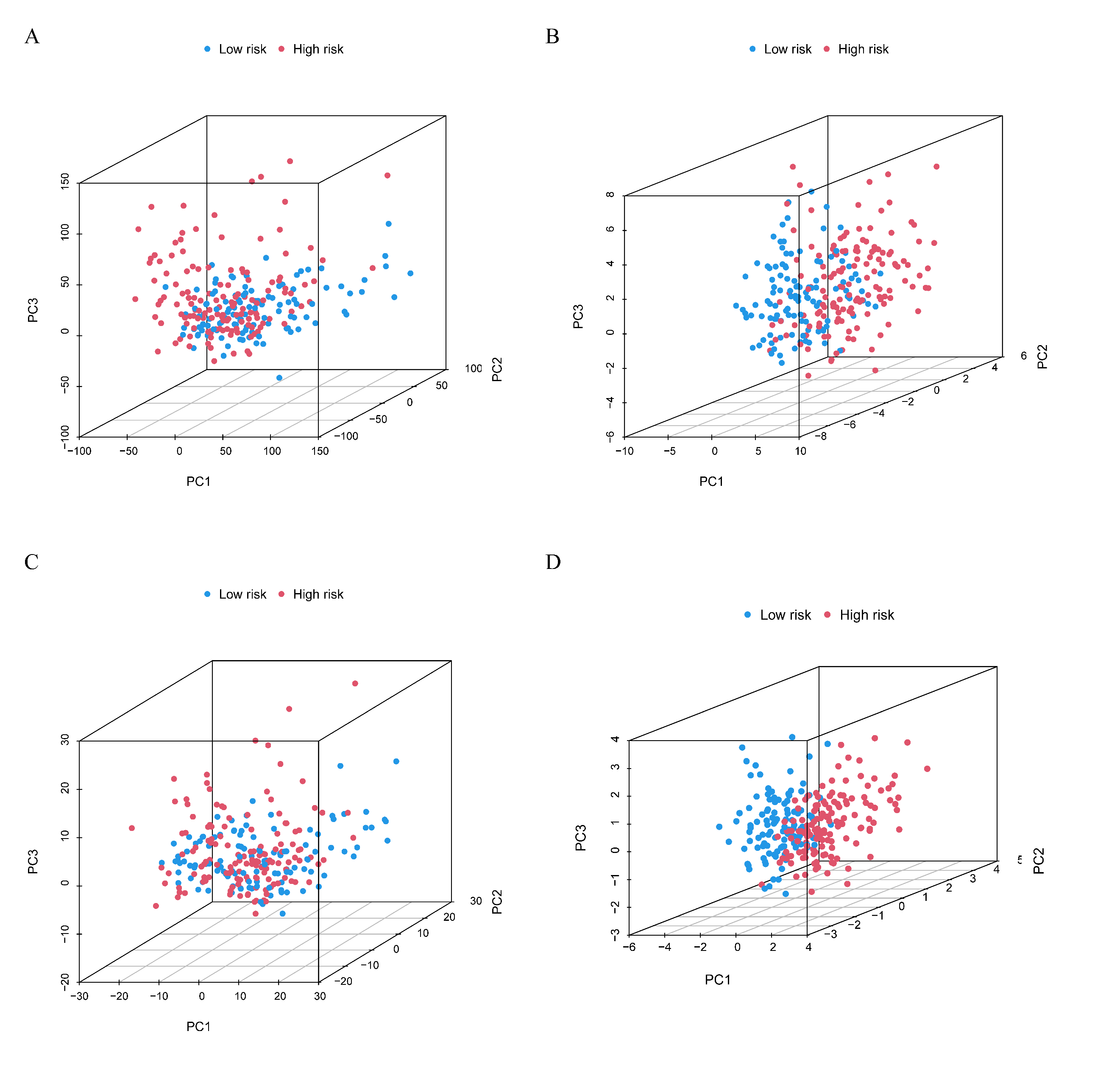
**

**Figure S3.** The result of PCA analysis. A. PCA of all genes; B. PCA of necroptosis genes; C. PCA of all necroptosis-related lncRNAs; E. PCA of the prognostic necroptosis-related lncRNAs.


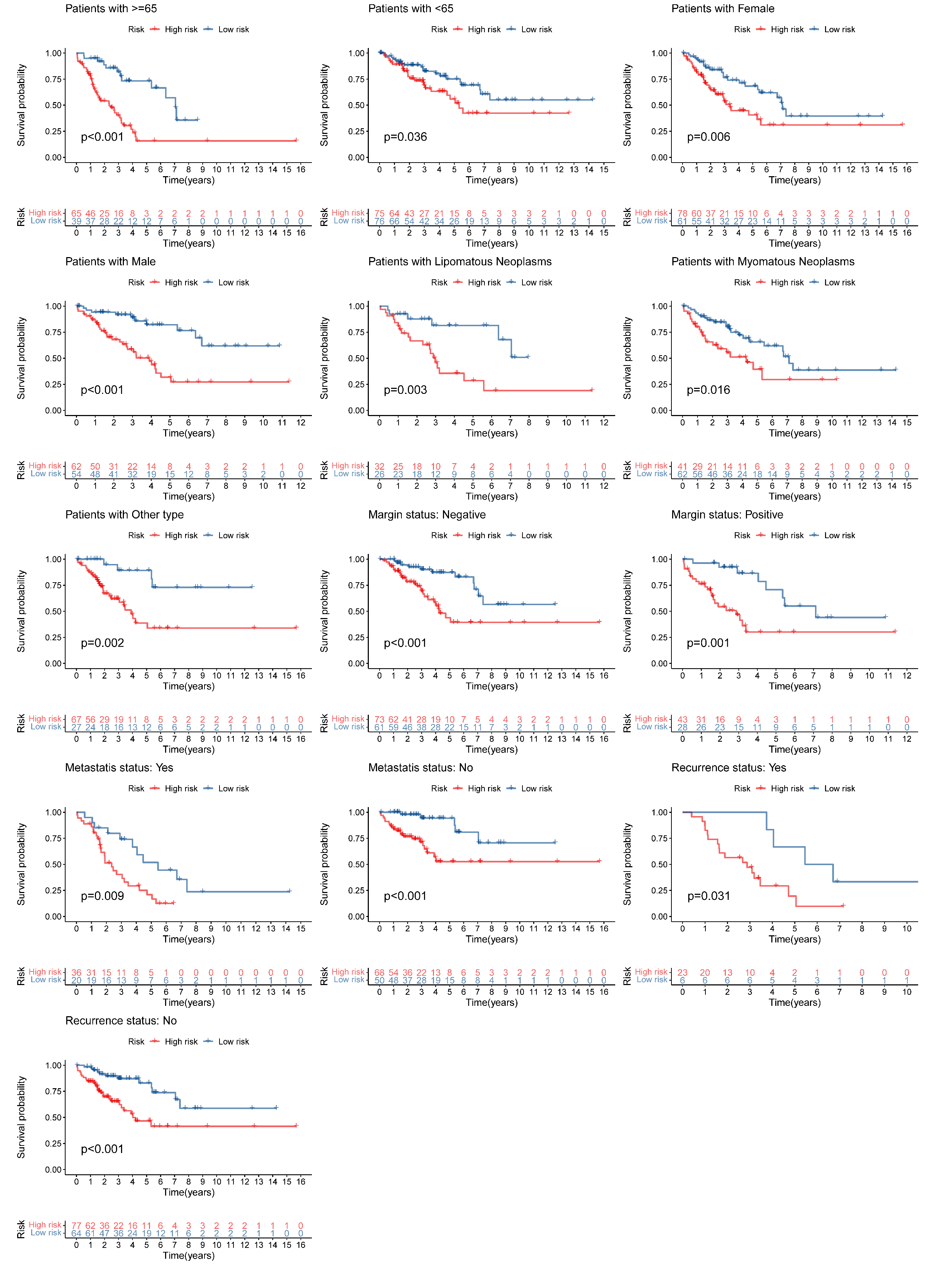


**Figure S4.** Kaplan–Meier survival curves of STS patients categorized by the clinical characteristic.


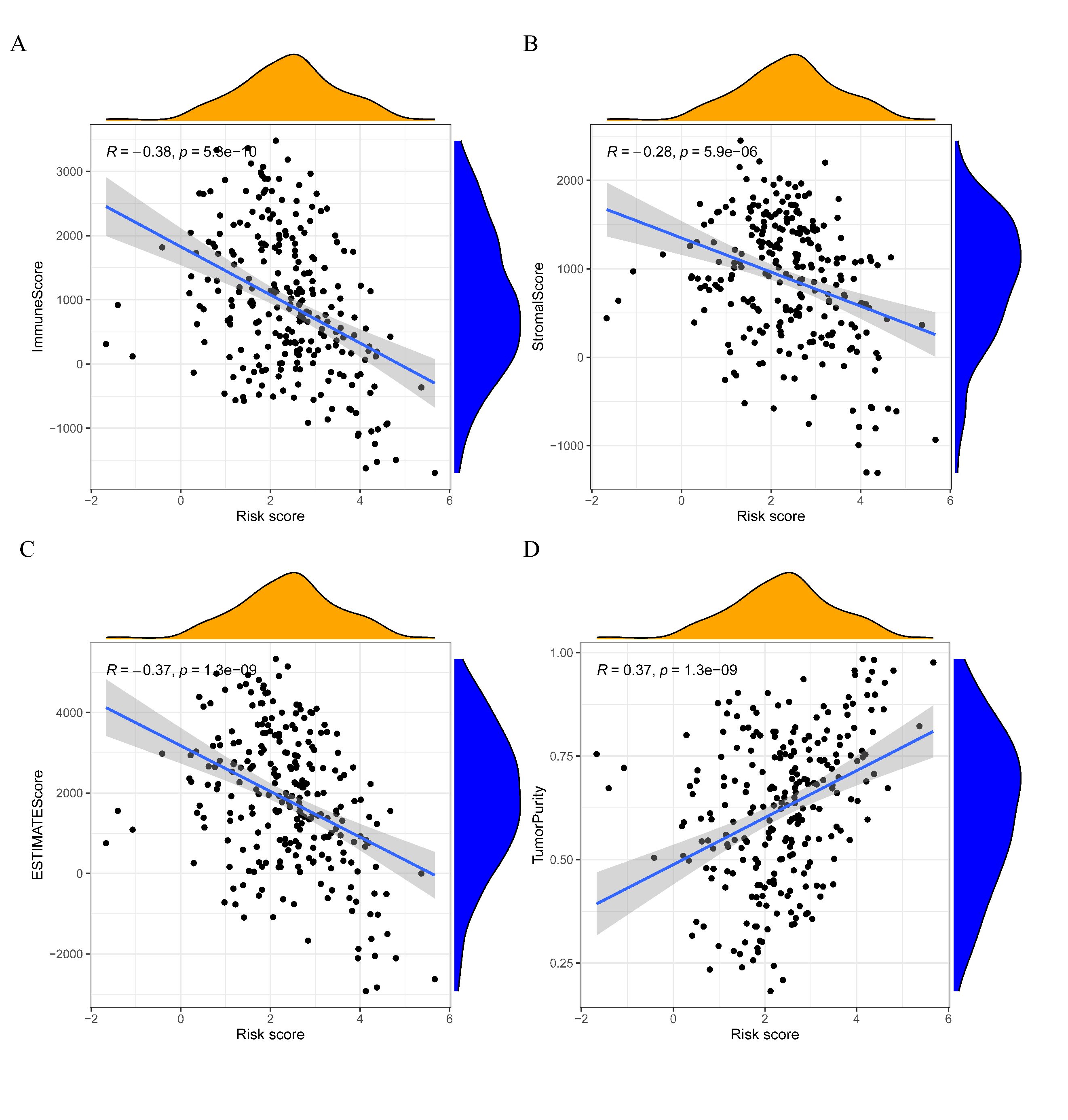


**Figure S5.** The association between risk score and tumor microenvironment score. A. Stomal score. B. Immune score. C. ESTIMATE score. D. Tumor purity score.

**
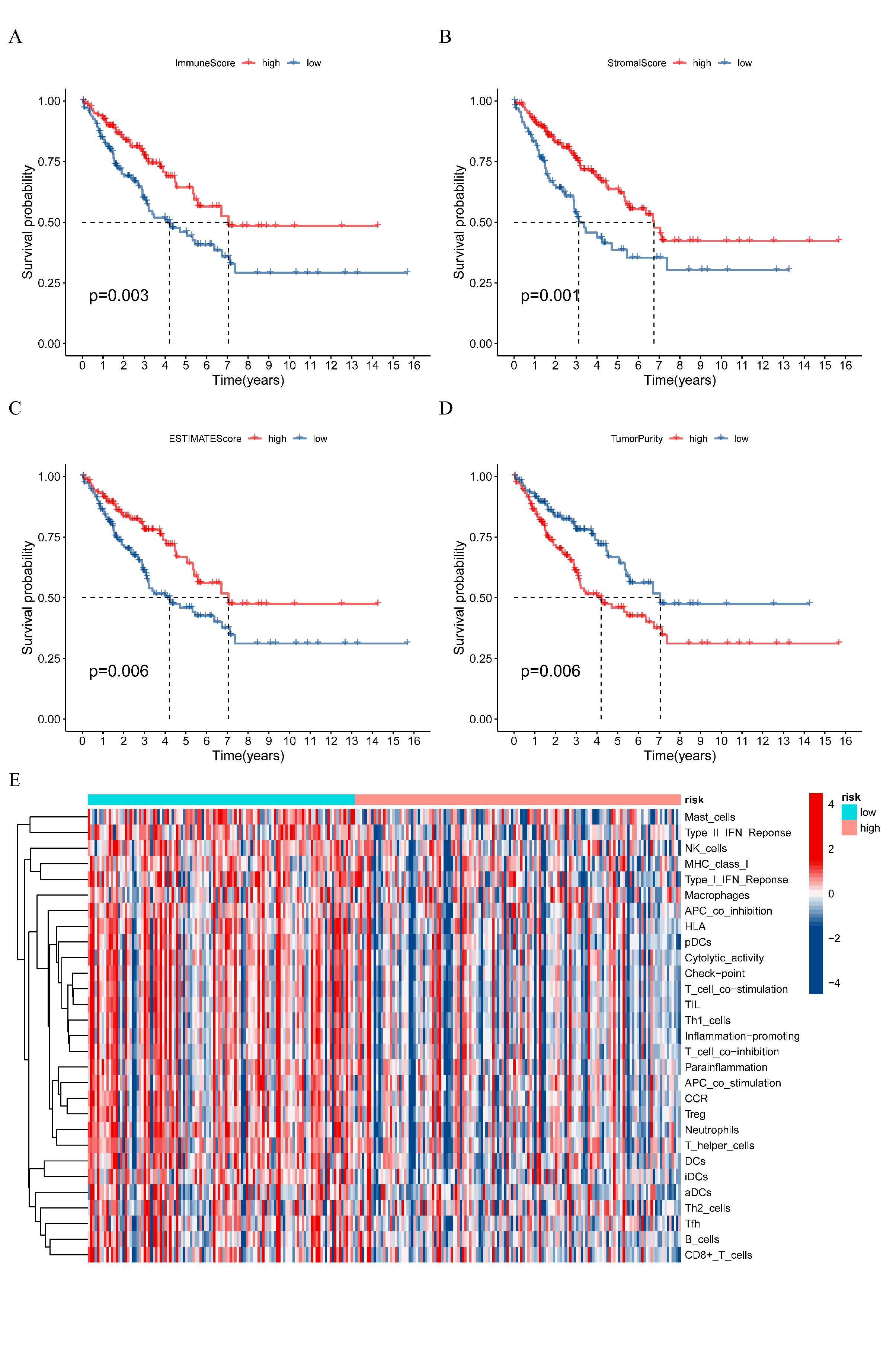
**

**Figure S6.** Kaplan–Meier survival curves of STS patients stratified by tumor microenvironment score. A. Stomal score. B. Immune score. C. ESTIMATE score. D. Tumor purity score. E. The heatmap for immune status based on ssGSEA.

**
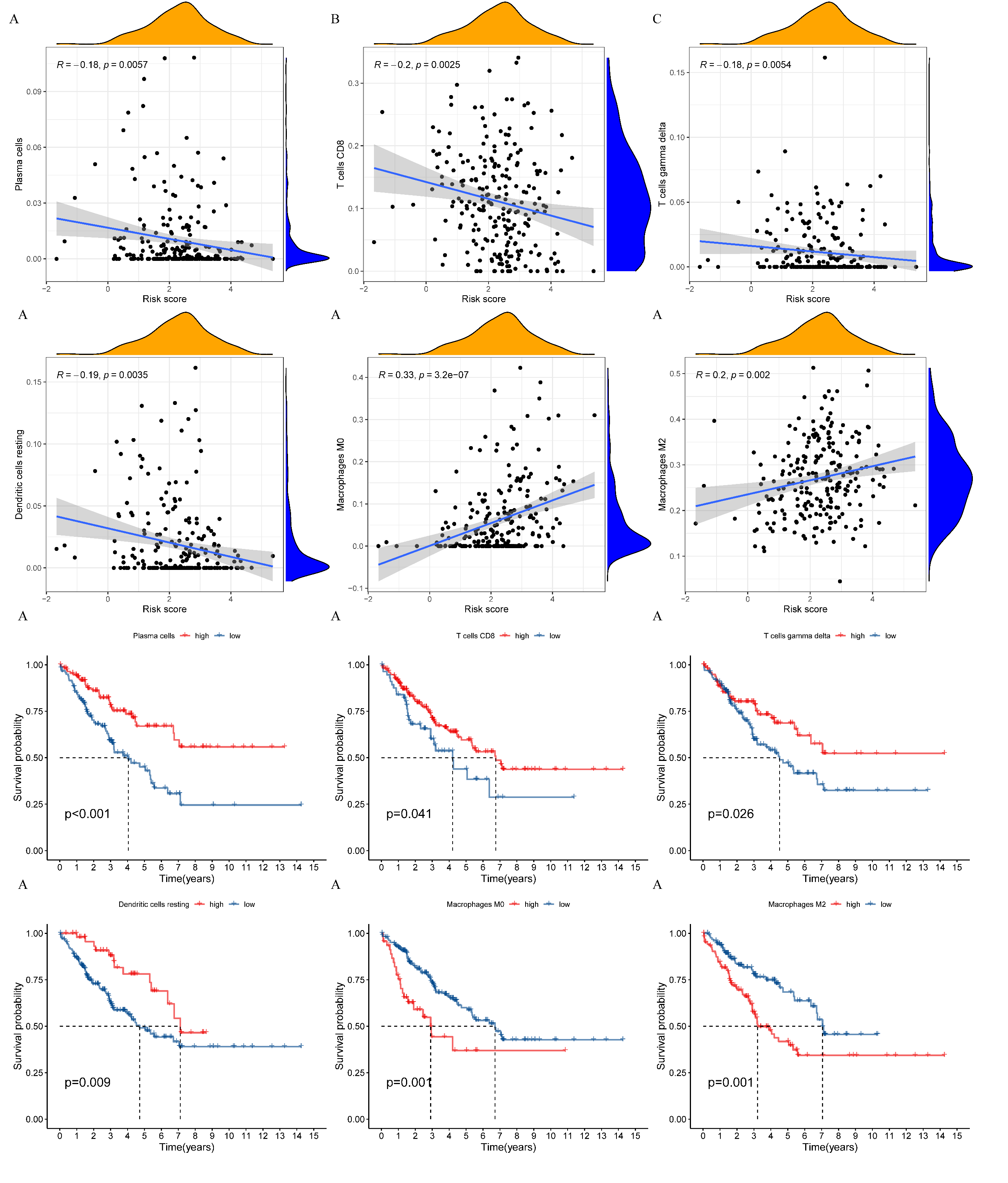
**

**Figure S7.** The relationship between immune cells and risk score. A. the correction between plasma cells and risk score; B. the correction between T cell CD8 and risk score; C. the correction between T cells gamma delta and risk score; D. the correction between Macrophages M0 and risk score; E. the correction between Macrophages M2 and risk score; F. the correction between Dendritic cells resting and risk score; G-L. Kaplan–Meier survival curves of STS patients stratified by plasma cells, T cell CD8, T cells gamma delta, Macrophages M0, Macrophages M2, and Dendritic cells resting.

**
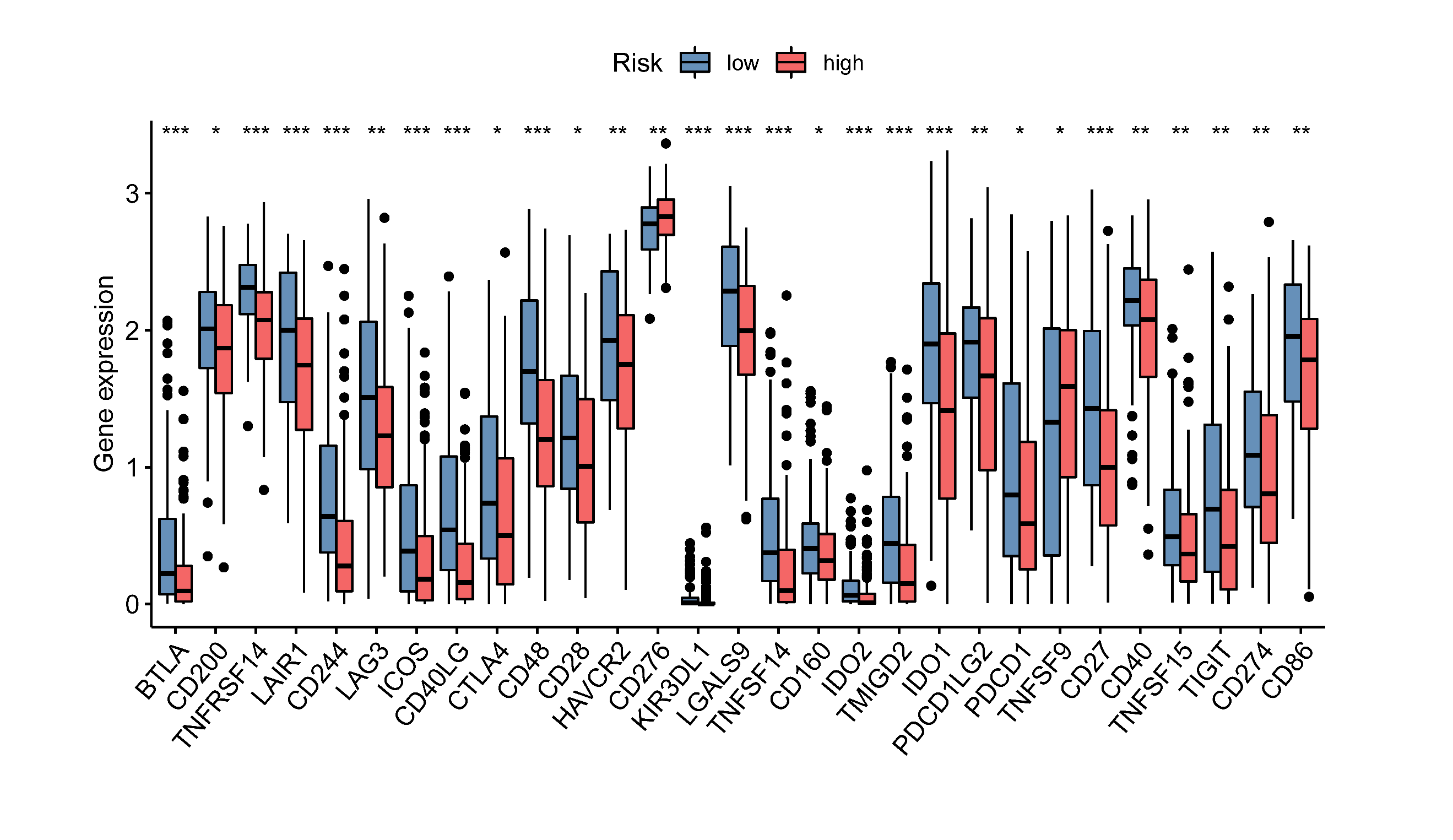
**

**Figure S8.** The difference of checkpoints expression in risk groups.
